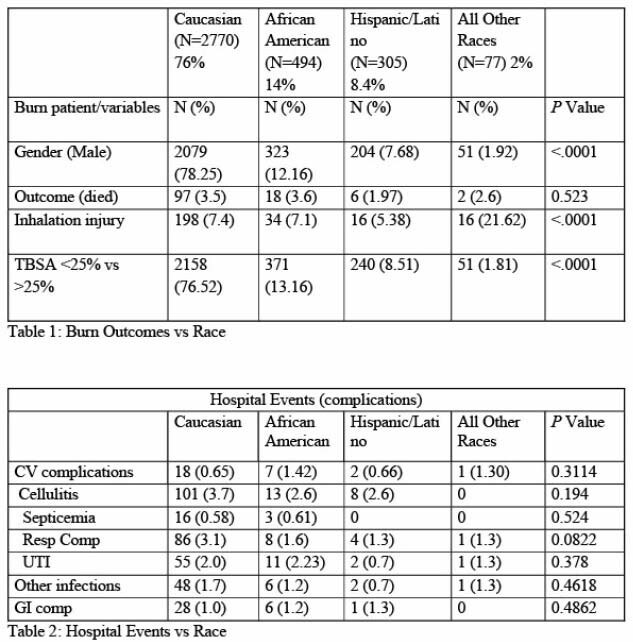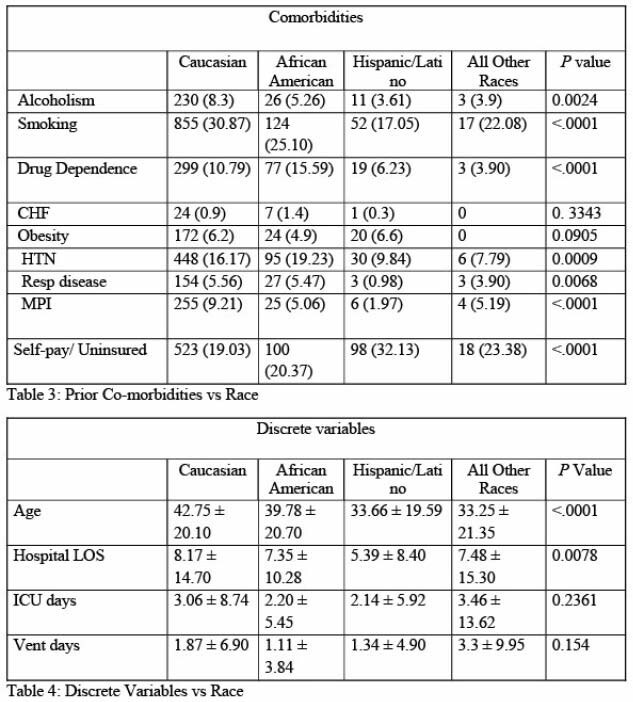# 898 Analysis of Healthcare Disparities in Burn Patients

**DOI:** 10.1093/jbcr/iraf019.429

**Published:** 2025-04-01

**Authors:** Rahel Eshete, Richard Korentager, Dhaval Bhavsar

**Affiliations:** The University of Kansas Health System Burnett Burn Center; University of Kansas School of Medicine; The University of Kansas Health System Burnett Burn Center

## Abstract

**Introduction:**

Prior research has indicated that race, gender, and socioeconomic status all influence the cost of care, outcomes, and mortality rates in burn patients. We wanted to see if there was any disparity in outcomes of our burn patients in relation to ethnicity and race.

**Methods:**

After IRB approval, we conducted retrospective review of burn registry data of 3646 patients from 2010 - 2020. We collected information on demographics, burn characteristics, co-morbidities, length of stay (LOS), number of surgeries, mortality, and complications. For the purposes of this study, the race category was divided into four groups: Caucasians, African American, Hispanic/Latino, and all others that include American Indian or Alaskan Natives, Asian, and Native Hawaiian or other Pacific Islander. The Hispanic/Latino group is categorized only if the chart selected that category for race and ethnicity.

**Results:**

We were able to validate data for 3646 patients for quantitative analysis. From these, 78% (N=2770) were Caucasian, 14% (N=494) were African American, 8.4% (N=305) were Hispanic/Latino and 2% (N=77) were other races. Mortality rates was 3.5% (N=97) for Caucasians, 3.6% (N=18) for African Americans and 1.97% (N=6) for Hispanic/Latino and 2.6% (N=2) for all other races with a p value of 0.523 suggesting no significant difference. Significant difference was identified for age (oldest for Caucasians); insurance status (lowest for the Hispanic/Latino group); co-morbidities (higher rate for smoking, HTN for Caucasians), length of stay (longest for Caucasians). Majority of the Caucasian group had TBSA < 25%, where as other race groups had variations of TBSA < 25%, 25 - 45%, 45 - 65% and > 65% (P value < 0.0001). There was no significant difference between the groups for complications and number of surgeries.

**Conclusions:**

There was no statistically significant difference in mortality rates between race groups. Caucasian group had longer length of stay which may be due to older age and higher comorbidities. Uninsured status did not adversely impact any of the outcomes including LOS for Hispanic group. It is possible to provide uniform care irrespective of racial and insurance status differences and verified burn center can be a model for it.

**Applicability of Research to Practice:**

This research was aimed to identify any institutional biases that were seen on a national study reflect similar findings in our institution. This study showed a holistic review of patient’s experience is imminent to providing the best care possible to diverse sets of population groups.

**Funding for the Study:**

N/A